# Deep learning can be used to train naïve, nonprofessional observers to detect diagnostic visual patterns of certain cancers in mammograms: a proof-of-principle study

**DOI:** 10.1117/1.JMI.7.2.022410

**Published:** 2020-02-04

**Authors:** Jay Hegdé

**Affiliations:** Augusta University, Medical College of Georgia, Departments of Neuroscience and Regenerative Medicine and Ophthalmology, Augusta, Georgia, United States

**Keywords:** deep learning, implicit learning, mammography, eye movements, representational similarity analysis, statistical learning, visual search

## Abstract

The scientific, clinical, and pedagogical significance of devising methodologies to train nonprofessional subjects to recognize diagnostic visual patterns in medical images has been broadly recognized. However, systematic approaches to doing so remain poorly established. Using mammography as an exemplar case, we use a series of experiments to demonstrate that deep learning (DL) techniques can, in principle, be used to train naïve subjects to reliably detect certain diagnostic visual patterns of cancer in medical images. In the main experiment, subjects were required to learn to detect statistical visual patterns diagnostic of cancer in mammograms using only the mammograms and feedback provided following the subjects’ response. We found not only that the subjects learned to perform the task at statistically significant levels, but also that their eye movements related to image scrutiny changed in a learning-dependent fashion. Two additional, smaller exploratory experiments suggested that allowing subjects to re-examine the mammogram in light of various items of diagnostic information may help further improve DL of the diagnostic patterns. Finally, a fourth small, exploratory experiment suggested that the image information learned was similar across subjects. Together, these results prove the principle that DL methodologies can be used to train nonprofessional subjects to reliably perform those aspects of medical image perception tasks that depend on visual pattern recognition expertise.

## 1 Introduction

Medical images are central to clinical decision-making in certain medical specialties, such as radiology and pathology. The complexities and ambiguities of the underlying images are known to be key contributing factors to diagnostic errors and to intra- and interobserver variability.^1^^–^^9^

Much progress has been made in understanding the perceptual and cognitive mechanisms that underlie clinical decision-making based on medical images.^10^^–^^31^ Nonetheless, we still do not have a quantitative understanding, especially that of a predictive value, of the underlying processes. For instance, we cannot measure, much less predict, the probability of a given diagnostic outcome given an individual medical image. Therefore, a rigorous understanding of what clinicians look for in the images and how they learn to look for it are crucial to reducing diagnostic errors, developing diagnostically assistive technologies, and improving patient outcomes and medical education.

In seeking to understand how radiologists and pathologists acquire and apply expertise in recognizing complex diagnostic patterns in medical images, it would seem most reasonable to directly test the clinicians themselves. However, this is not always possible, or even desirable. For one thing, it has been widely recognized that there are a number of practical difficulties in testing, say, diagnostic radiologists in sufficient numbers (see, e.g., Refs. 22 and 31). For another, testing fully trained experts is not necessarily the best way of understanding how the expertise is acquired in the first place. In addition, a substantial body of previous research has demonstrated the validity and usefulness of testing certain aspects of medical image perception in nonprofessional subjects^31^ (also see Ref. 32). But in order to test anything but the simplest aspects of medical image perception, the subjects will have to be trained, to one degree or another, in performing tasks based on image patterns. However, there are relatively few such methods that are currently available. The present study seeks to help fill this gap.

We have previously shown that naïve subjects can be trained to become experts in complex visual pattern recognition by adopting the principles of a type of machine learning called “deep learning” (DL), where the subject learns the task-relevant statistical properties of complex images using a set of suitably labeled training images.^33^^–^^38^ Of course, DL is widely used to train machines to perform a variety of real-world tasks, including complex visual pattern recognition.^39^^–^^43^ It has been shown that, using comparable methods, pigeons can be trained to reliably detect the diagnostic visual patterns of certain cancers, such as microcalcifications, in medical images.^44^ DL has functional similarities to the well-known perceptual learning phenomenon called implicit learning, where viewers, including human infants, learn task-relevant properties of images even when they are not explicitly instructed as to what to learn.^45^^–^^50^

In the present study, we sought to extend our aforementioned studies of human deep learning^33^^–^^38^ to the context of medical images. That is, we sought to establish the principle that DL can be used to also develop expertise in recognizing visual statistical patterns diagnostic of cancer in medical images in naïve, nonprofessional subjects, using mammography training as an exemplar case. To this end, we chose, as our exemplar cases of cancer, breast cancers associated with microcalcifications and breast masses. In these cancers, the cancerous tissue tends to have characteristic visual patterns (see, e.g., breast images in Figs. 5–8) that can be diagnostic of cancer at a comparatively high level.^53^^–^^59^ This has made breast masses and microcalcifications breast cancer image features of choice in many previous cognitive and computational studies, including those involving computer-assisted detection/diagnosis systems (see, e.g., Refs. 14–17 and 60–64) and psychophysical studies of breast cancer detection (see, e.g., Refs. 20 and 65–67). Since the overall goal of this study was simply to prove the above principle—namely, that naïve subjects can learn the diagnostic statistical properties of medical images from suitably “labeled” examples—we chose these advisedly and admittedly simple and straightforward classes of images as the exemplar cases in our study (see Sec. 4 for caveats).

The main (i.e., first) experiment described below demonstrates that DL can indeed be used to develop such expertise in recognizing such diagnostic image patterns of cancer. We show, using rigorous, well-established methods of signal detection theory (SDT)^68^^,^^69^ that, upon being trained to criterion, subjects were able to classify, at a highly statistically significant level, mammograms with cancer versus mammograms without cancer. By this standard functional SDT definition, the trained subjects were able to reliably detect cancers in our mammogram set, in which the diagnostic visual patterns were highly salient. We wish to make explicit at the outset that this is not necessarily to say that other visual patterns of breast cancers can be similarly learned or that this is all there is to detecting breast cancer in a clinical setting (for more on these and other caveats, see below).

Also in the main experiment, we explored whether and to what extent the subjects’ eye movements, especially microsaccades, change in a learning-dependent fashion. We focused on microsaccades because they are associated with high-acuity visually guided behavior, e.g., when the subjects scrutinize a given image region or attend to it (for recent reviews, see Refs. 70–74), such as those that are likely to occur when subjects scrutinize mammograms. We also describe three additional exploratory experiments that test the efficacy of various potential enhancements to the DL technique and to characterize the various phenomenological aspects of this DL effect. Some of the preliminary results of this study have been previously reported in abstract form.^75^^–^^77^

## 2 Methods

### 2.1 Subjects

All subjects who participated in this study were adult volunteers with normal or corrected-to-normal vision and had no prior training or experience in any field of medicine, including those involving medical images. All subjects who were between the ages of 18 and 65 years of age, had normal or corrected-to-normal vision, and agreed to participate in the study were enrolled in the study. No subject who met these eligibility criteria was excluded. All subjects gave written informed consent prior to participating in the study. All procedures related to study subjects were approved in advance by the Institutional Review Board (IRB) of Augusta University, where the experiments were carried out.

A total of 29 different subjects participated in this study. Of these, 14, 11, 4, and 4 subjects participated in experiments 1–4, respectively. All four subjects who participated in experiment 4 also participated in experiment 2 (i.e., were previously trained to criterion using the paradigm in exp. 2 prior to participating in exp. 4). Four subjects withdrew from the study before completing their participation. These subjects are excluded from the subject counts above, and the data from these subjects are not included in this study in compliance with our IRB-approved protocol.

### 2.2 Experiment 1: Basic Deep Learning Paradigm

**Fig. 1:**
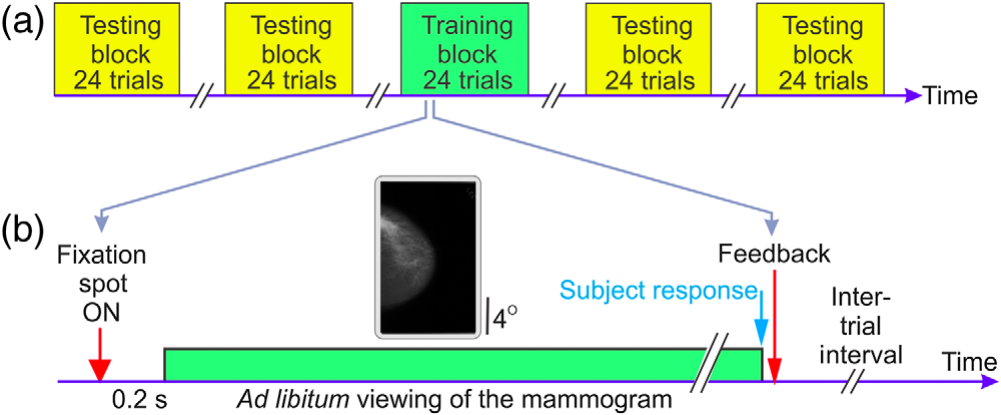
Design of experiment 1 (main experiment). (a) The experiment consisted of a variable number of training blocks (green rectangles) preceded and followed by testing blocks (yellow rectangles). (b) Trial paradigm during the training blocks. The trial paradigm during the testing blocks was identical, except that subjects received no feedback (not shown). Not drawn to scale. See text for additional details.

The experiment consisted of three successive phases: a pretraining test phase followed by a training phase, followed in turn by a post-training test phase [Fig. 1(a)]. The trials during each phase were carried out in blocks of 48 trials each. The pretraining and post-training phases consisted of two blocks of trials each. The training phase consisted of a variable number of blocks, depending on how many blocks it took for the subject to reach criterion performance. To maximize subject comfort, subjects were allowed unlimited breaks in between trial blocks and individual trials (also see below).

#### 2.2.1 Stimuli

For reasons noted above, we focused on mammograms as a proof-of-principle case (also see Sec. 4). Also for reasons noted above, we focused on mammograms with two classes of cancer: microcalcifications and breast masses.

All mammograms used in this study were obtained from the the Digital Database for Screening Mammography (DDSM) public database.^78^^,^^79^ All mammograms in this database are radiologically vetted and have known ground truths as to the cancer status of the given mammogram. In most cases (except for mammograms classified as “normal,” which we did not use), professionally demarcated region/s of mammographic interest (ROI/s) of the mammogram are also available. Each breast is available in two standard views of screening mammography, craniocaudal (CC) and mediolateral oblique (MLO), in this database.^78^^,^^79^

We selected noncancerous mammograms from those classified as “benign” (i.e., negative [−ve] for cancer), so labeled because they were ambiguous enough to necessitate patient callback, but were eventually determined to be benign.^78^^,^^79^ This is the category of noncancer mammograms in this database whose appearance was most similar to that of cancer mammograms in the database. We screened the mammograms in this category against the following two criteria: (i) the mammogram contained exactly one ROI and (ii) the narrowest aspect of the ROI was at least 200 pixels wide. We selected a total of 632 unique mammograms and 316 unique breasts (given that each breast was imaged from CC and MLO views; see above) that met both of these criteria. We then similarly screened the mammograms classified as “cancer” (i.e., positive [+ve] for cancer), and selected another set of 632 mammograms that also met both of the above criteria and a third criterion in this case that the ROI in question had either microcalcification or breast mass, but not both. Of these 632 mammograms, 401 (63%) had microcalcifications, and the remaining ones had breast masses. No systematic differences between these two classes of cancer were evident in any of our experiments (not shown). For this reason, we pooled the results from the two classes. Alphanumeric markings on the mammograms, when present, were digitally masked for all mammograms.

Prior to the start of the experiment, 48 stimuli (a random 24 with cancer and another random 24 without cancer) were set aside for use as stimuli during the testing blocks (see below). These stimuli were not used during the training blocks, thus ensuring that the training versus testing used mutually nonoverlapping stimulus sets. Thus in order to perform significantly above chance levels, the subjects had to learn the task-relevant statistical properties of the stimuli and could not rely on familiarity with or memory of, if any, previously encountered stimuli.

Prior to each block of trials, the above stimulus set was randomly reshuffled and 24 unique images (corresponding to 12 unique breasts, each in CC and MLO views) each from the benign and cancer categories were drawn. Stimuli were presented on a neutral gray screen at a resolution of 800×600  pixels at 60 Hz projected on to a tangent screen using an SVGA projector (Epson Inc.) at a luminance of $20\;{\mathrm{cd}/m}^{2}$. The primary motivation for using this stimulus presentation system was to make it compatible with our high-speed eye tracker (see below) and as comparable as possible to the system used in our magnetic resonance imaging (MRI) scanner for future neuroimaging studies and to the system used in our earlier studies on learning to recognize camouflaged objects (or camouflage-learning)^34^ that motivated the present study. However, using this system required a down-sampling of the mammograms, which was implemented by scripts custom-written in Presentation,^80^ which was used for stimulus presentation, experimental control, and data collection.

#### 2.2.2 Task paradigm

During each trial of the training phase, subjects performed a two-alternative forced choice cancer detection task with feedback. Each trial began when the subject fixated on a central fixation spot and pressed a key to indicate trial readiness. The rationale for requiring central fixation was to ensure that the eyes started out at the same position across all trials. Provided the subject maintained fixation for the next 200 ms (as determined by high-resolution eye tracking, see below), a mammogram randomly drawn as described above was presented for *ad libitum* viewing. Subjects were required to indicate whether or not the mammogram contained a cancer by pressing a designated button on the computer’s mouse, upon which the stimulus was turned off and a positive audio feedback was presented if the subject’s response was correct. Subjects received no feedback upon making an incorrect response, so that the lack of a positive audio feedback served to indicate to the subjects that their response was wrong. We chose this response design because subjects expressed strong preference for it in pilot studies. Note that our feedback regime provided the subject with an implicit “labeling” as to whether the preceding mammogram had a cancer or not.

Whenever the subjects were ready for the next trial, they initiated the trial by pressing a key as described above. Such opportunities for *ad libitum* breaks in between trials, in addition to the aforementioned opportunities to take *ad libitum* breaks in between trial blocks, allowed subjects to learn at their individual pace, which we have found to be effective in the aforementioned camouflage-learning studies.^34^ As an empirical matter, subjects initiated the next trial after an average intertrial interval of 1.9 (±0.3  SEM) s.

Note that the sole explicit task requirement was to report the presence or absence of a cancer in the given image. The only information that the subjects received during the training phase was the visual information in the images, and the feedback following correct responses. Subjects were not told what to learn or how to learn it. Thus, our training paradigm met the functional criteria of human DL.^37^

A subject was deemed to have reached the criterion level of learning when he/she performed at discriminability or $d^{\prime }\geq 1.35$ (corresponding to hit and false-alarm rates of ∼0.75 and ∼0.25, respectively, and p<0.05, for Gaussian data^68^^,^^69^^,^^81^^–^^83^) for ≥3 consecutive training blocks on the same day.

The trials during the testing phases were identical, except that subjects received no feedback of any kind.

Monocular eye position of the subjects was monitored continuously at 1 kHz throughout the entire block using EyeLink II high-speed video eye tracker (S-R Research, Ottawa, Ontario, Canada). The eye tracker was recalibrated at the beginning of every block.

Data were analyzed using programs custom written in R (R Development Core Team^84^), MATLAB (Mathworks, Natick, Massachusetts), or Python. Cancer detection performance was measured using a variety of conventional and signal detection-theoretic measures^68^ for each trial block individually as previously described.^34^ Microsaccades were analyzed using both R and MATLAB.

### 2.3 Experiment 2: Deep Learning with Image Review

This experiment was identical to experiment 1, except as follows. Eye position was not monitored during this experiment. Subjects were asked to fixate the central fixation spot as they indicated trial readiness at the start of each trial, but the fixation requirement was not enforced. Stimuli were presented on a higher resolution monitor (1440×2560  pixels; 60 Hz; Dell Inc.), and the subjects made their responses using a game pad (Logitech Inc.). Subjects performed the same cancer detection task as before [“Cancer detection task” stage of the trial; see Fig. 6(a), left]. After the subjects made their response and received the feedback (if any), subjects were given an opportunity to review the same mammogram, but with the outline of the radiologically vetted ROI digitally superimposed on the mammogram [“Image review” stage; see Fig. 6(a), right]. The motivation for this design was our previous finding, from our comparable prior psychophysical studies of deep learning,^33^^,^^34^^,^^76^ that subjects learned better if they were given an opportunity to re-examine the image in light of the feedback.

### 2.4 Experiment 3: Image Review in Light of Diagnostic Information

This experiment was identical to experiment 2, except as follows: During the review phase, radiologically vetted diagnosis and diagnostic information was also presented along with the mammogram with the ROI outlined, so that subjects could re-examine the mammogram in light of this additional information (see Fig. 7).

### 2.5 Experiment 4: Representation Similarity Analysis

#### 2.5.1 Stimuli

##### Stimulus set

The stimulus set consisted of a total of 32 partial view mammograms (PVMs) generated by digitally clipping the mammogram so as to fully encompass the radiologically vetted ROI, as we have described in detail before in Refs. 51 and 52 [also see Figs. 8(a) and 8(b)]. Of these, 16 were +ve for cancer and 16 were −ve for cancer. Out of these, only a randomly selected 4 PVMs (2 +ve for cancer and 2 −ve for cancer) were tested, with 64 repetitions for each possible pair, for each subject. The rationale for testing only a random subset was that testing the entire set of 32 PVMs would require a large number of trials per subject, even when each pair of stimuli is tested only twice, and only the off-diagonal lower (or only the upper) triangle of the response dissimilarity matrix (RDM; see below) is estimated: For a stimulus set with n=32 stimuli and number of repetitions r=2, a total of {[(n*n)−n]/2}*r=992 trials.

#### 2.5.2 Task paradigm

We have previously described our representational similarity analysis (RSA) task paradigm in detail.^52^ Briefly, during each trial, subjects viewed a given pair of PVMs *ad libitum* (Fig. 8). Subjects reported, using an on-screen slider, how perceptually dissimilar the two mammograms were. The rationale for requiring the subjects to report the perceived dissimilarity as opposed to the perceived similarity is that it ultimately makes the underlying analyses more principled.^85^ Briefly, RSA works by determining how far apart visual percepts (or internal representations) are in an abstract mental space. In this space, identical percepts are expected, by definition, to have zero distance between them. It follows that the more dissimilar two images, the more dissimilar resulting internal percepts, and larger distances between the perceptions in the abstract space. This naturally allows the subject to decide how to “put a number on” the perceived dissimilarity, i.e., scale the perceived dissimilarities himself or herself. It is ultimately for this reason of allowing the subjects to self-scale that it is advisable to have them report dissimilarities than similarities.^86^^–^^89^

Each possible pair of PVMs was presented at least twice in randomized order, with the stimulus location (left versus right) swapped between repetitions, and the dissimilarity ratings were averaged across repetitions [Ref. 52; also see Figs. 9(b) and 9(d) and Ref. 51].

#### 2.5.3 Data analysis

We constructed a perceptual RDM P, wherein cells P(i,j) and P(j,i) represented the average reported perceptual dissimilarity between a given pair of PVMs i and j [see, e.g., Figs. 9(b) and 9(d); also see Ref. 52]. We similarly constructed a separate RDM S for stimuli, where S(i,j) and S(j,i) represented the physical dissimilarity (measured as 1-cophenetic correlation^97^^,^^98^) between a given pair of PVMs i and j. To relate the viewer’s internal representations to the sensory information in the PVMs, we quantitatively compared matrices P and S using established methods,^85^^,^^88^^,^^99^ including the congruence coefficient^85^ [see Fig. 9(f)]. Briefly, the congruence coefficient C is a principled method for measuring the similarity between two given matrices of the same size (in our case, two 4×4 matrices S and P). Essentially, it calculates the pairwise Euclidean “distance” between every given cell of S to every given cell of P, and averages and normalizes the distances to generate a single value of C for a given pair of matrices. Values of C can vary between 0 (denoting two completely different matrices) to 1 (two identical matrices). The statistical significance of C can be straightforwardly measured using standard randomization methods.^52^^,^^85^

## 3 Results

### 3.1 Experiment 1 (Main Experiment): Naïve Subjects Can Learn to Detect Certain Cancers in Mammograms

The main goal of this experiment was to determine if our previously described DL training methodology can be successfully extended to training naïve, nonprofessional subjects to reliably detect cancer in medical images. Note that this meant that numerous training parameters were substantially different from those typically used in clinical settings (including, but not limited to, screen resolution; see Sec. 2 for details). Since optimal DL training requires a large number of training images with known ground truths, and since screening mammograms (i.e., mammograms used for periodically testing otherwise asymptomatic women for breast cancer) meet this criterion, we chose them as exemplar medical images in our study.

Our experiments used a block design, whereby we obtained a baseline measure of task performance using testing blocks before training, followed by as many training blocks as were needed to train a given subject to criterion, followed again by post-training testing blocks [Fig. 1(a)]. Within each block, subjects performed a randomly shuffled series of trials and received feedback during the training blocks [see Fig. 1(b)], but not during the testing blocks. Thus in this experiment, subjects had to learn to detect cancer in mammograms (i.e., successfully classify each given mammogram as +ve or −ve for cancer, by the aforementioned signal detection-theoretic operational definition) they had just viewed and the ensuing feedback they had received. No other task-relevant information was available to them.

As expected from the fact that our experimental design specifically allowed for each subject to learn the task at his/her own pace, the number of trial blocks needed for a given subject to train to criterion levels ($d^{\prime }\geq 1.35$) varied considerably across subjects (mean, 24.7 blocks; median, 22.3 blocks; range, 12 to 39 blocks).

**Fig. 2:**
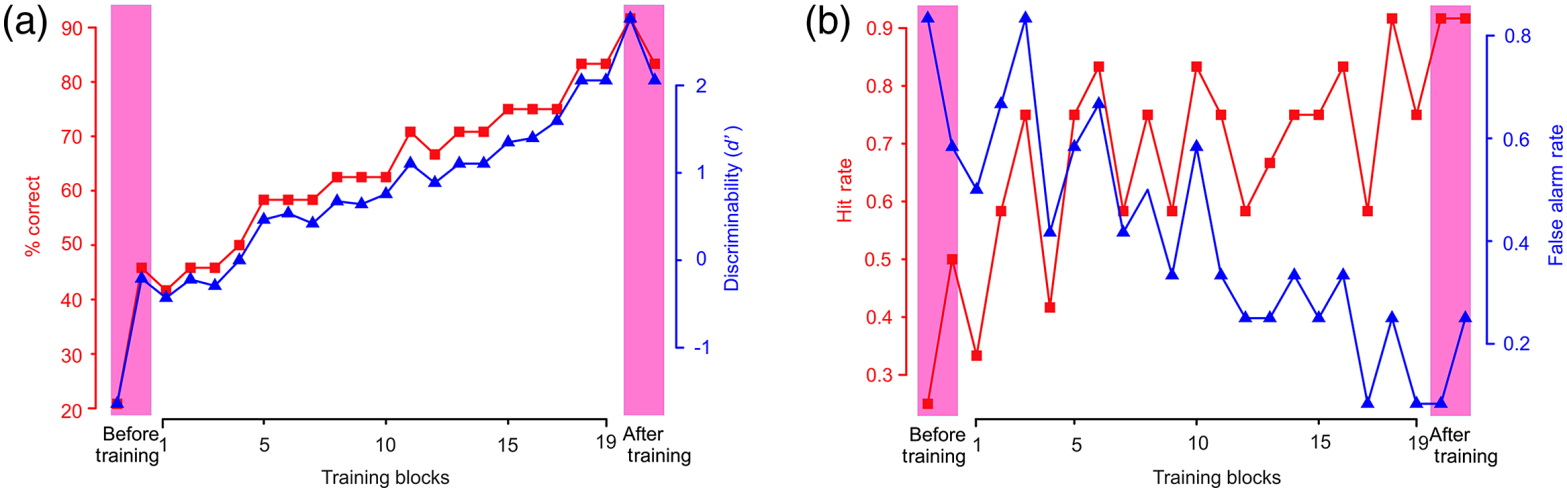
Performance metrics of an exemplar subject (subject 01-07) before, during, and after training in experiment 1. In this figure and multiple other figures in report, two sets of color-coded data, with the corresponding color-coded y axis labels on either side, are plotted in each panel. In each plot, individual data points represent a single scalar value calculated for each block, and therefore do not have error bars. (a) Task performance measured as percent of correct responses (red plot and y axis on left) and $d^{\prime }$ (also known as discriminability;^69^ blue plot and y axis on right). (b) Hit (or true positive) rate and false alarm (or false positive) rate.^69^

For clarity, we will first illustrate key results using the data from an exemplar subject in experiment 1, and then present the results averaged across the entire subject sample. Figure 2 shows the performance of an exemplar subject in experiment 1. The subject’s task performance, measured both as percent correct and as discriminability ($d^{\prime }$), was statistically indistinguishable from chance during the testing blocks before training [Fig. 2(a), pink highlight at far left], indicating that the subject was unable to perform the task without training, i.e., the task was not trivially easy. During the training blocks, the performance steadily improved and remained at comparable levels during the post-training testing, suggesting continued feedback was not needed to sustain the performance [Fig. 2(a)]. The trend in both metrics was statistically significant for this subject [Mann–Kendall (MK) trend test for $d^{\prime }:\;z=5.51$, p<0.05; MK test for percent correct: z=5.12, p<0.05].

Figure 2(b) shows the breakdown of the $d^{\prime }$ data into its two components, hit rate (or sensitivity) and false alarm rate. The relative trends in the two components show that the improvements in $d^{\prime }$ earlier in the training came primarily from a decrease in false alarms (wherein the subject incorrectly reported a noncancerous breast as cancerous), and that the increases in hits (wherein the subject correctly reported a cancerous breast as such) played a more prominent role in the improvements in the task performance later in the training. The explanatory intuition for these results, consistent with our observations (also see Fig. 5 below), is that at the very beginning of the training, the subjects do not know anything about the underlying data, but know that the task requires them to identify those images that contain a cancer. Thus, understandably enough, they reported mammograms with visually salient parts, such as breast densities [see, e.g., Figs. 5(a) and 5(b)] as cancerous (although many of these cases are not cancerous), thus resulting in high false alarm rates initially. Learning that high-salience parts *per se* are not reliably diagnostic of cancer and learning what is takes time, which is reflected in comparatively slower improvements in hit rates.

Together, these results provide a quantitative picture of the temporal dynamics of this learning phenomenon in this individual subject, and help empirically illustrate the larger truism that the overall learning-dependent changes in the performance in any subject reflect a complex interplay of many of these factors.

#### 3.1.1 Learning-dependent changes in eye movements: microsaccades

The overall motivation behind monitoring eye movements in this experiment was to quantitatively characterize and document training-dependent changes, if any, in eye movement patterns. As alluded to above, we focused on microsaccades, because they represent fixational eye movements that result when the viewer is actively scrutinizing a region of the image that is of interest to the viewer (see Refs. 70–74). Moreover, our preliminary results in a related previous experiment had suggested that the number of microsaccades decreases as the subjects get better at the cancer detection task.^77^ We therefore deemed it useful to determine if this effect is reproducible in the current experiment and, if it is, document this potentially important phenomenology of DL. A related motivation was that microsaccades, by virtue of being associated with visual scrutiny, may help future studies to determine what exactly is learned in DL and how it is learned, both of which remain shrouded in mystery (also see below).^37^^,^^43^^,^^100^

**Fig. 3:**
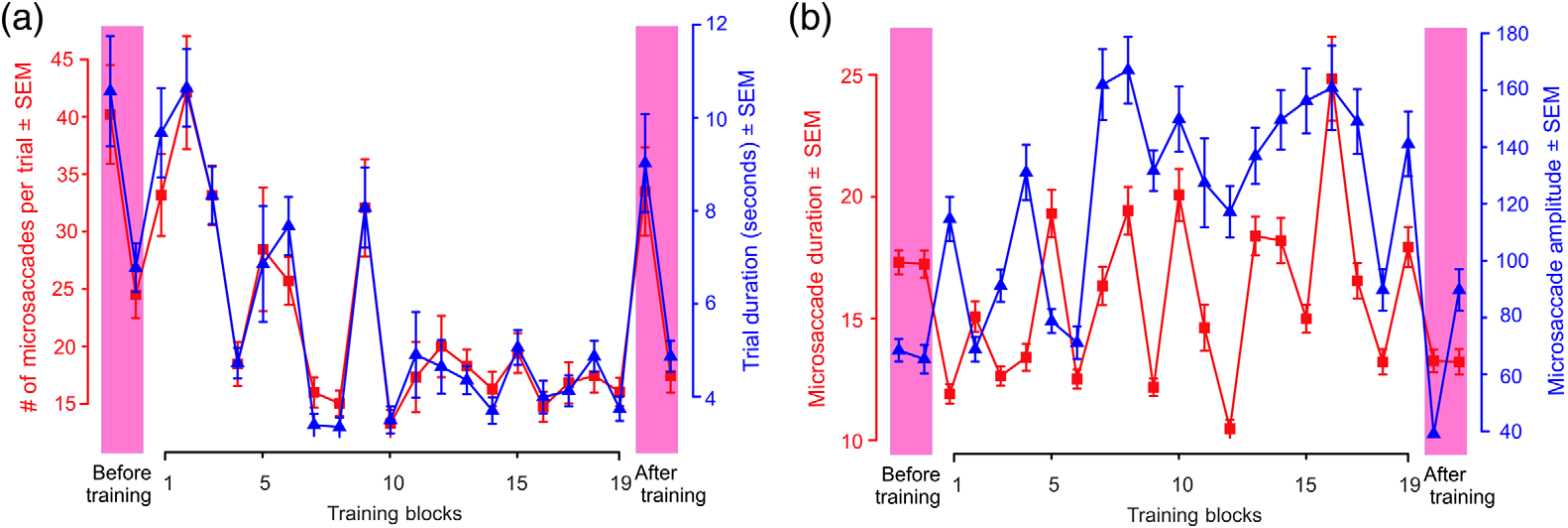
Microsaccades of an exemplar subject (subject 01-07; same subject as in Fig. 2) before, during, and after training in experiment 1. (a) Number of microsaccades during each trial (red plot and y axis on left) and duration of the (self-paced) trials (blue plot and y axis on right). (b) Duration of microsaccades (red plot and y axis on left) and the amplitude of microsaccades (blue plot and y axis on right). In both plots, error bars denote standard errors of the mean across trials during each given run.

Figure 3 shows the learning-dependent changes in eye movements, specifically microsaccades, in the same exemplar subject as above [see Sec. 2 for procedural details; also see Figs. 5(a) and 5(b)]. The number of microsaccades during a given trial systematically decreased for this subject throughout the training [Fig. 3(a), red plot]. This metric was highly correlated with the trial duration [Fig. 3(a), blue plot; r=0.89, df=20, p<0.05]. Thus, across the training blocks, the subject’s performance generally improved (i.e., the subject’s decisions got more accurate), even as the subject took less time and made fewer microsaccades before reaching the decision. The duration and amplitude of microsaccades also showed modest learning-associated upward trends for this subject [Fig. 3(b)].

#### 3.1.2 Changes in performance across all subjects

**Fig. 4:**
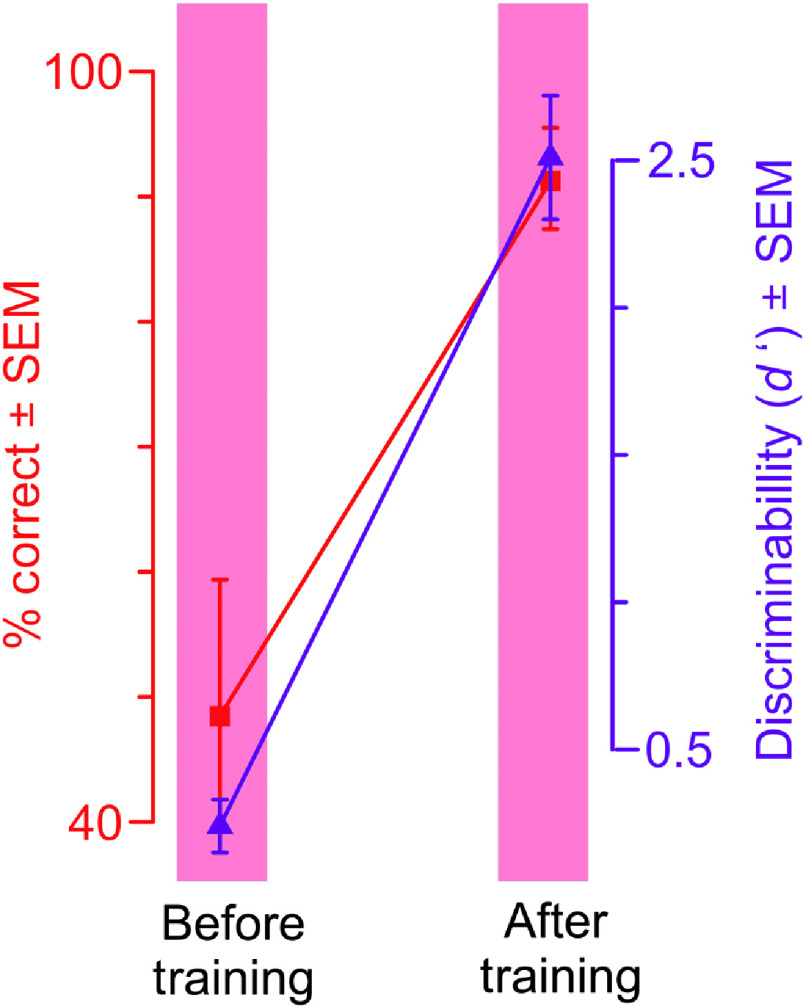
Training-dependent changes in task performance across all subjects in experiment 1. Averaged performance (subject-to-subject SEM) across all subjects (N=14) before and after the training are shown (left and right columns, respectively). Data corresponding to two metrics of task performance metrics are shown: percentage of correct trials (red symbols and the y axis to the left) and discriminability (i.e., or d prime; blue symbols and the y axis to the right). The data from the training blocks are excluded from this figure, because different subjects required varying number of training blocks to reach this level, so that averaging training block data across subjects were uninformative at best.

The 14 subjects who participated in this experiment each achieved criterion level performance. The performance of these subjects before and after the training is shown in Fig. 4 (see legend for details). Three subjects who enrolled in this experiment voluntarily withdrew while the training was ongoing, and the data from those subjects are excluded from this figure and from this report at large (also see Sec. 4.2).

The training-dependent improvement in performance as measured by $d^{\prime }$ was significant across all subjects and for each individual subject, corrected for multiple comparisons [Tukey’s honestly significant difference (HSD) test, p<0.05]. This also held when performance was measured using the percentage of correct trials, instead of $d^{\prime }$ (Tukey’s HSD test, p<0.05). Together, these results demonstrate that our DL training paradigm can be used to train naïve, nonprofessional subjects to reliably detect cancer in mammograms.

#### 3.1.3 Training-dependent changes in microsaccades

**Fig. 5:**
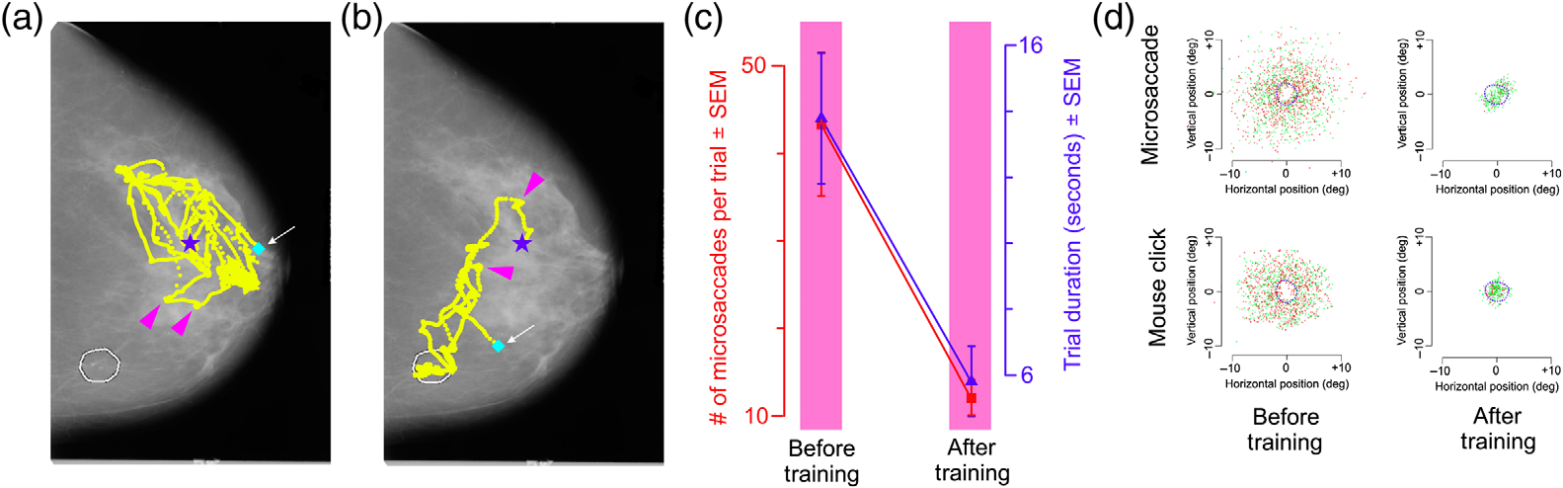
Learning-dependent changes in microsaccade patterns in experiment 1. (a) Eye movements of an individual subject (subject 01-12) during a single trial in the pretraining testing block (subject’s $d^{\prime }$ during the block=0.12, p>0.05). (b) Eye movements of a different subject (subject 01-05) elicited by the same mammogram during a single trial in the post-training testing block (subject’s $d^{\prime }$ during the block=2.39, p<0.05). In both panels (a) and (b), the irregular outline at bottom left denotes the radiologically determined location of cancer in this mammogram; the blue star denotes the starting eye position during each trial; the colored diamond denotes the eye position at the time of the response; and the pink arrowheads denote selected microsaccades. Note that, in both panels, the response (small white arrow) occurred sometime after the last microsaccade, indicating that the subjects were not scrutinizing anything in particular when they responded. (c) Microsaccade patterns across all subjects (N=14) before and after the training. (d) A subset of the subjects (N=3) in this experiment was asked to indicate the location of the cancer, if any, in the given mammogram using a mouse click. This panel shows the spatial patterns of microsaccades (top row) and mouse clicks (bottom row) in these subjects. Each plotting symbol denotes data from a single microsaccade or mouse click during a single trial, and the position of the symbol denotes the distance (measured in degrees of visual angle), where 0 represents the center of the radiologically vetted ROI. Green and red symbols denote correct and incorrect trials, respectively. The dotted blue circle denotes the mean outline of the ROI averaged across all relevant mammograms.

The training-dependent reductions in the microsaccade frequency, i.e., number of microsaccades per trial, noted anecdotally above for one subject, also held across all subjects (Fig. 5). Visual examination of the eye movement patterns of subjects during each individual trial (not shown) indicated that before training, microsaccades were directed at visually salient portions of the mammogram, and improved performance was associated with a tendency to fixate image regions that were more diagnostic of cancer, which were often less visually salient. This effect is illustrated anecdotally by the typical eye movements elicited by the same mammogram before and after the training for two different subjects [Figs. 5(a) and 5(b); see legend for details].

Across all subjects, the number of microsaccades per trial and the trial duration both decreased significantly upon training [Fig. 5(c); Tukey’s HSD tests, p<0.05 in each case]. This indicates that in general, subjects took less time and made fewer microsaccades before reaching the decision as their performance improved across training blocks. This experiment did not seek to address the causal relationship between the improvements in the performance on the one hand and the changes in microsaccade patterns on the other. Further studies are needed to resolve this intriguing issue.

Figure 5(d) (top row) illustrates the training-dependent changes in the locations of microsaccades. For visual clarity, only the location of the microsaccade immediately prior to the response [see Figs. 5(a) and 5(b) for the rationale] is plotted in these figures relative to the center of the radiologically vetted ROI (dotted blue outline). Before training [Fig. 5(d), top left], the subjects seldom fixated these ROIs, as evidenced by the fact that relatively few microsaccades landed within the ROI [dotted circle in Fig. 5(d), top left; see legend for details]. This is arguably because, as noted above, the subjects tended to fixate visually salient regions of the mammogram before the training. After the training, the subjects tended to fixate the ROI immediately prior to responding [Fig. 5(d), top right]. The same overall effect held when the subjects were asked to use a mouse click to localize the cancer [Fig. 5(d), bottom row]. Together, these results indicate microsaccades got more infrequent, and tended to occur nearer to the ROIs, as the performance improved during the training, suggesting that the subjects became more efficient in searching the mammogram for cancer. Our experiment did not address the mechanisms by which visual search becomes more efficient in this fashion; further studies are needed to address this question.

### 3.2 Experiment 2. Opportunity for Review Improves Training Outcomes

In experiment 1 above, the subjects did not have an opportunity to revisit the image they had just viewed so as to determine what they got right or wrong in light of the feedback. However, in our prior DL studies in other contexts, we have found that opportunity to re-examine the visual image in light of the feedback improved learning outcomes, in that subjects reached the criterion faster, and at higher levels.^33^^–^^38^

**Fig. 6:**
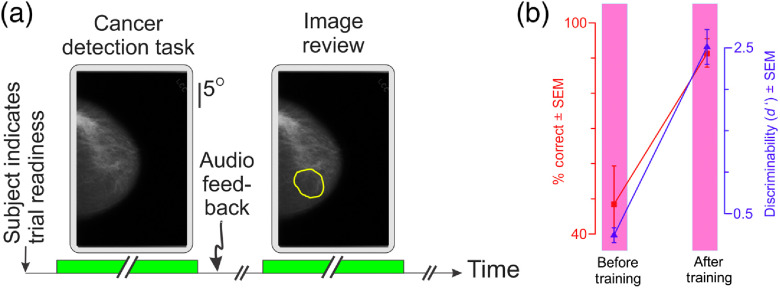
Task paradigm of, and results from, experiment 2. (a) A typical trial during the training block. See text for additional details. (b) Task performance across all subjects (N=11) before and after the training plotted using the same plotting conventions as in Fig. 4.

Experiment 2 sought to determine whether this effect was reproducible in the present context. In this experiment, subjects (N=11) were provided an opportunity to re-examine the mammogram in light of the ground truth [Fig. 6(a); see legend for details]. Subjects required about one-third fewer training blocks to reach criterion levels (mean, 14.9 blocks; median, 13 blocks; range, 10 to 27 blocks; Welch 1-tailed t test, t=2.48, df=20.22, p<0.05), and reached significantly higher levels of performance [Fig. 6(b); paired, one-tailed t test, t=8.08, df=10, p<0.05]. These results suggest that providing the subjects an opportunity to revisit their decisions may result in better outcomes. However, it should be noted that our results by no means prove this notion, in part because the experiment was not designed to address whether the changes in learning outcomes, if any, are solely attributable to the change in the training paradigm. Future studies, perhaps using a randomized controlled study design, are needed to address this issue.

### 3.3 Experiment 3. Opportunity for Reviewing Decision Plus Diagnostic Information Improves Training Outcomes

**Fig. 7:**
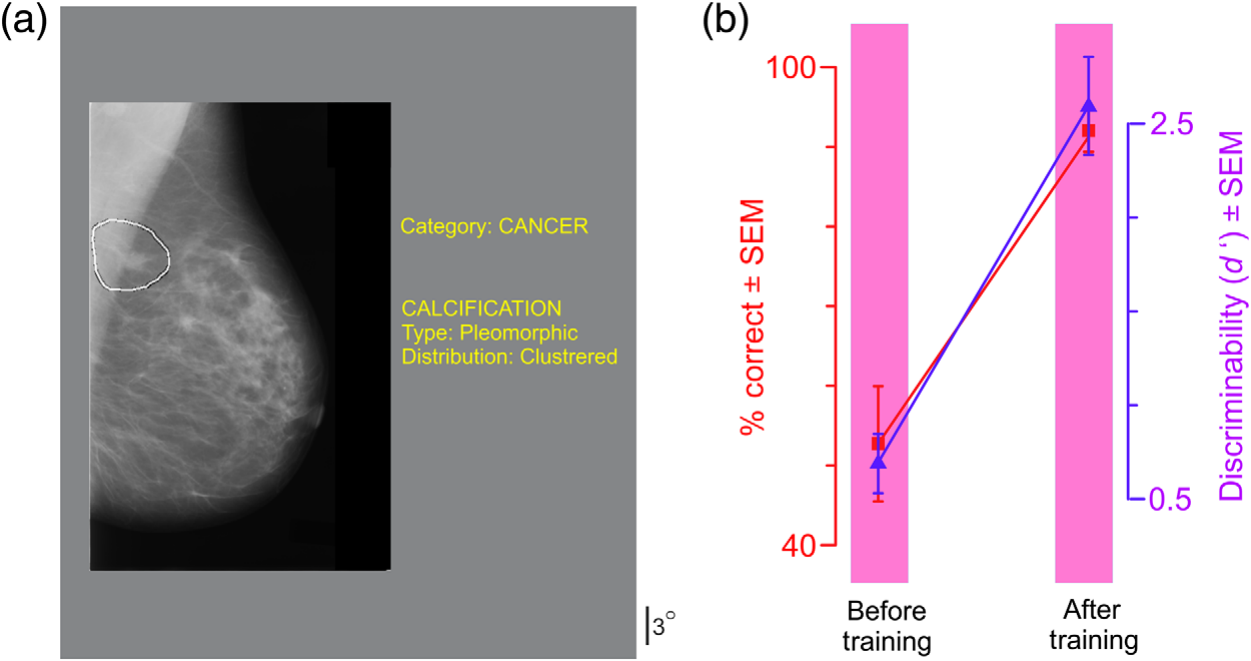
Experiment 3. (a) During the Image review stage of each trial, the radiologically vetted diagnosis and the diagnostic info (text in yellow), along with the mammogram with the outline of the ROI, was presented for *ad libitum* viewing. See text for details. (b) Task performance across all subjects (N=4) before and after the training plotted using the same plotting conventions as in Fig. 4.

This experiment was identical to experiment 2 above, except that we also provided radiologically vetted diagnosis and diagnostic information during the image review stage following the subject’s response [Fig. 7(a); see legend for details]. Technical terms in the diagnostic information (e.g., “pleomorphic”) were explained to the subjects in simple terms with the aid of an illustrated introductory textbook on mammography.^101^

We found that this paradigm also resulted in faster learning compared to experiment 1, in which subjects required about 40% fewer training blocks to reach criterion levels (mean, 10.0 blocks; median, 10.5 blocks; range, 9-22 blocks; Welch 1-tailed t test, t=2.34, df=7.96, p<0.05), and reached significantly higher levels of performance [Fig. 7(b); paired, one-tailed t test, t=6.93, df=3, p<0.05]. Once again, all the caveats noted in the context of experiment 2 above also apply to interpreting this result.

### 3.4 Experiment 4: Representational Similarity Analysis Can Be Useful for Characterizing Some Aspects of Deep Learning

The foregoing experiments demonstrate that DL methods can, in principle, be used to train naïve, nonprofessional subjects to detect diagnostic image patterns of breast cancer in mammograms (also see Sec. 4). This straightforwardly raises the question of what it is that the subjects deep-learn in mammograms and whether and to what extent different subjects learn the same patterns. This is an extremely difficult question to experimentally ascertain, because it requires one to somehow measure the internal mental representations of subjects. On the other hand, the need for approaches to addressing this issue is especially keen in the context of DL, because in DL subjects are not told what to learn, and it remains possible that each subject learns his/her own idiosyncratic visual patterns.

Fortunately, RSA, a rigorous, ingenious method in mathematical psychology developed by Roger Shepard starting in the 1950s,^86^^–^^88^^,^^102^ can be used to address this issue. We have previously demonstrated the usefulness of RSA in a related context, i.e., that of determining, in principle, whether different radiologists have similar mental representations of diagnostic features of cancer in mammograms.^52^ In this experiment, we sought to establish the feasibility—again, in principle—of using RSA to help address the learning of diagnostic features of cancer in mammograms by naïve subjects.

**Fig. 8:**
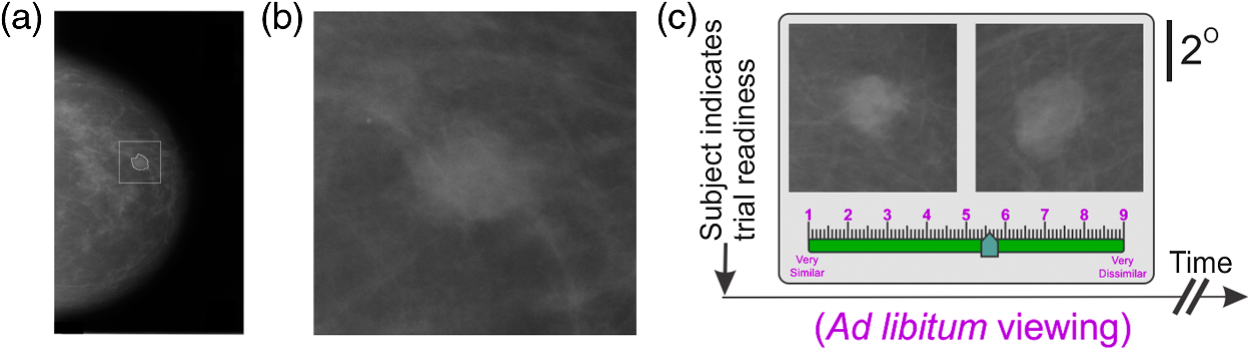
Task paradigm used in experiment 4. (a) A whole-breast mammogram (WBM). (b) An image fragment, or “PVM” generated from the WBM in panel (a). The irregular outline in panel (a) denotes the radiologically vetted ROI, and the square denotes the image region cropped from the mammogram to generate the PVM shown in (b). The fact that “distracting” information is minimized in PVMs makes them much more tractable psychophysically and computationally. We therefore used PVMs rather than whole breast mammograms in this experiment.^51^ (c) The dissimilarity rating paradigm used in exp. 4. Trials were self-paced by the subject, so that each trial started when the subject indicated trial readiness (far left) by pressing a key on the computer’s keyboard. After a short (100 ms) gap, the subject was presented a pair of PVMs for *ad libitum* viewing. The subject was required to make a graded report of the perceived dissimilarity between the two images (using an on-screen slider, bottom), and press a separate key (not shown) to confirm the report. Figure not drawn to exact scale. For additional details, see Ref. 52.

**Fig. 9:**
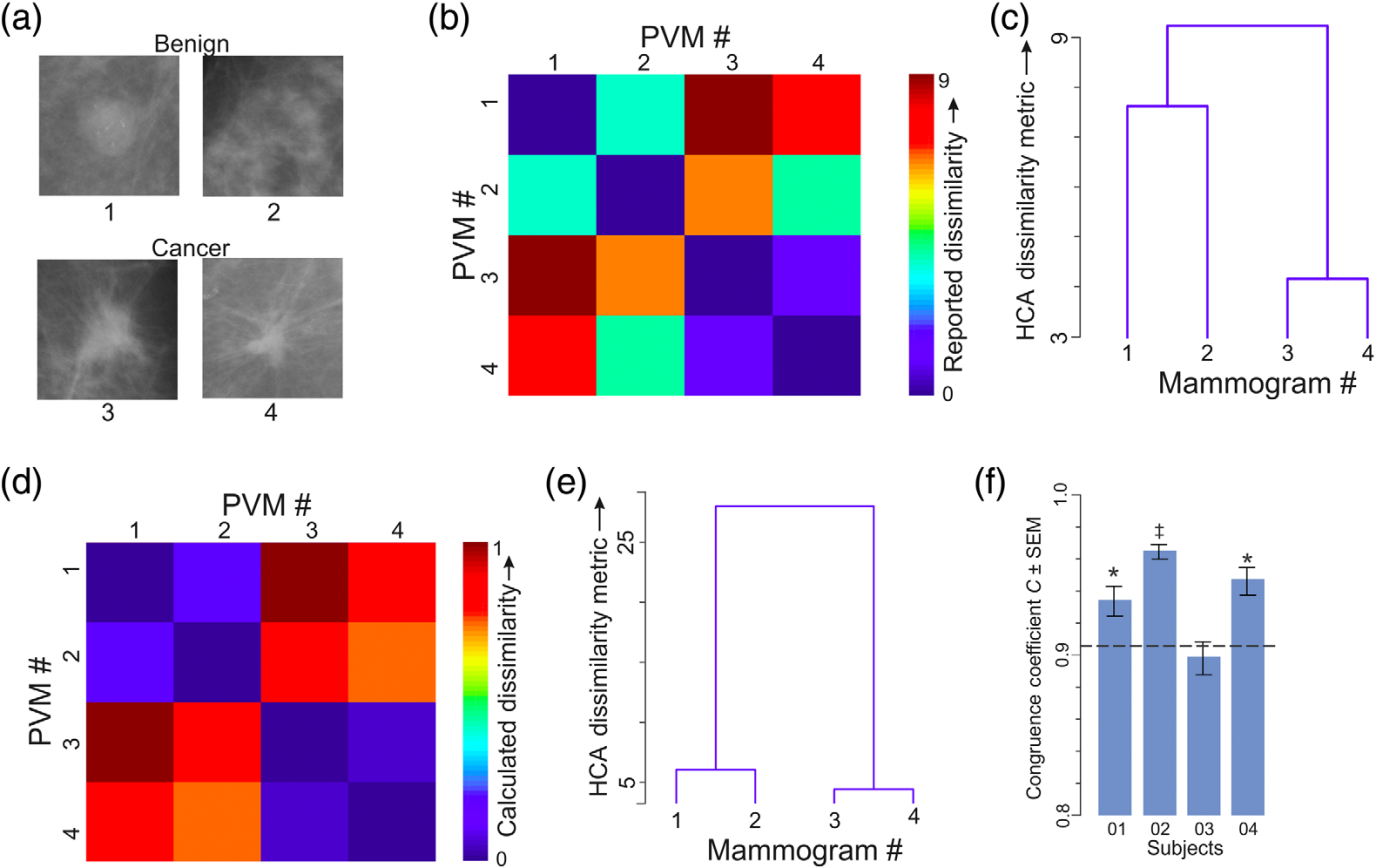
Results of experiment 4: similarity between the physical features of mammograms versus their internal representations in four trained nonprofessional subjects. (a) 579 radiologically vetted PVMs (with 329 +ve PVMs with a single cancer; 177 with microcalcification and the rest with breast mass) were generated as described in Figs. 8(a) and 8(b). Of these, 32 randomly selected PVMs [16 of which were +ve for cancer (9 with microcalcification and 7 with breast mass) and 16 of which were −ve for cancer] were used as stimuli in exp. 4. Four representative PVMs (2 −ve, top row and 2 +ve, bottom row) are shown in this panel. The PVM numbering in this panel is used in panels (b)–(e). (b), (c) The perceptual RDM P for a single nonprofessional subject (subject 04-02) trained to criterion. Data are averaged across 64 repetitions for each stimulus pair and are shown in heatmap and hierarchical cluster analysis^90^^–^^95^ dendrogram formats in panels (b) and (c), respectively. Note that the differences between +ve and −ve stimuli are magnified in this trained subject. (d), (e) The corresponding stimulus RDM S of the four mammograms was calculated as described in Sec. 2. Note that the differences between +ve and −ve stimuli, measured by the vertical distances between stimuli in the dendrogram,^90^^,^^92^^–^^96^ are rather subtle, indicating that the +ve and −ve stimuli were physically quite similar. (f) Similarity between P and S as measured by the congruence coefficient^85^ for four different trained nonprofessional subjects in this experiment (subject 04-02 is denoted by the second bar from left). The dotted line denotes the significance threshold for this dataset, as determined by randomization.^85^ *, p<0.05; ‡, p<0.01.

To this end, we quantitatively compared the internal mental representations of the mammograms in nonprofessional subjects (N=4) after they were trained to criterion using the paradigm in experiment 2 (for Sec. 2 for procedural details; also see Ref. 52). Briefly, subjects viewed mammogram fragments or PVMs that contained the ROI [see, e.g., Fig. 9(a)]. The rationale for using PVMs rather than whole breasts is to help ensure that the subjects carried out the task based on the ROI rather than some other aspect of the breast [see Figs. 8(a) and 8(b) for additional details]. Subjects rated the PVMs with respect to how dissimilar in appearance they were (see Ref. 52 and Sec. 2 for procedural details). The subjects’ pairwise ratings were arranged in the form of a perceptual RDM P as we have described before in Ref. 52. We similarly constructed an RDM S that captured the physical, pairwise dissimilarity among the PVMs. We quantitatively compared the RDMs from different subjects using the established methods of RSA as described in Sec. 2.^88^^,^^103^^,^^104^

Figure 9(a) shows one subset of four PVMs used for each subject. Each subject was similarly tested with seven other, mutually nonoverlapping subsets of PVMs (not shown). Figure 9(b) shows the perceptual RDM P for the four PVMs in Fig. 9(a) for one exemplar subject trained to criterion. Figure 9(c) displays the same data in P in a different format, i.e., as a conventional hierarchical clustering plot, where the vertical distance between a given pair of PVMs denotes how dissimilar the subject perceived them to be. Note that in both Figs. 9(b) and 9(c), this trained subject perceived cancer PVMs (PVMs #3 and 4) to be highly similar, even though they were far from physically identical. Similarly, the subject perceived PVMs #1 and 2 (both benign) to be similar. But note that this subject reported cancer PVMs as a group to be highly dissimilar to the benign PVMs as a group. This illustrates, for this PVM subset, the given subject’s internal mental representations of cancer versus benign visual patterns can be quantitatively summarized using RSA.

How do these internal representations of this subject correspond to the sensory image patterns of cancer versus benign? To answer this, we first calculated the pairwise physical similarity of the PVMs (see Sec. 2) and generated the sensory RDM S for the same set of 4 PVMs [Figs. 9(d) and 9(e)]. Note that this matrix S is a function of each stimulus set; it does not vary from one subject to the next. Note that the cancer versus benign PVMs are physically quite similar [Fig. 9(e)], but the cancer versus benign differences are considerably exaggerated in the internal representation of the trained subject. This suggests, although by no means proves, that DL may work by exaggerating the small physical dissimilarities. More importantly, RSA can help provide potential insights such as this into the seemingly intractable cognitive processes that underlie DL.

To measure the extent to which the subject’s internal mental representation of the image patterns for this PVM set corresponds to the actual physical patterns, we calculated the congruence coefficient C for this particular pair of P and S. (Note that the fact that the corresponding cells in the two matrices have substantially different absolute values does not matter; it is the relative pattern of the values across the matrices that matters. That is, one of the many advantages of RSA is that it provides a scale-invariance at every stage, which makes it possible to compare quantities as different as those represented in P and S.) As alluded to in Sec. 2, higher values of C denote greater similarity between P and S.

We repeated this procedure for each of the eight possible nonoverlapping subsets of the PVMs noted in Sec. 2 for each subject, and calculated the C for each of the eight rounds for each subject. Figure 9(f) shows the average C value (±SEM) for each subject. For three of the four subjects, the C values were statistically significant (see legend for details), indicating that for these subjects, the internal perceptual representations corresponded significantly with the physical image patterns. These results also suggest, given the aforementioned fact that the values of S are the same for a given PVM set for all subjects, at least three out of four subjects perceived similar image patterns from a given set of PVMs. The reason/s for the lack of significant congruence in subject 03 [see Fig. 9(f)] is unclear; our sample sizes were, out of the practical necessities noted in Sec. 2, too small to rule out a lack of sufficient statistical power. Indeed, caveats related to small sample sizes apply to all conclusions from this study.

Altogether, the results of RSA suggest, albeit by no means prove, that different subjects learn comparable diagnostic patterns from their DL training. More broadly, this experiment proves the general utility of RSA in addressing some of the most important questions about how DL works in human subjects.

## 4 Discussion

Collectively, our results demonstrate that principles of DL can be used to train naïve, nonprofessional subjects to reliably detect certain cancers in screening mammograms (see below for important caveats). Our results also suggest that providing the subjects an opportunity to re-examine the mammograms in light of the feedback and additional diagnostic information resulted in better learning outcomes. Results of RSA indicate that the learned visual patterns were likely similar across subjects. That is, different subjects are likely to have learned similar visual patterns from similar mammograms.

### 4.1 Relationship to Previous Studies and the Novelty of the Present Study

As noted above, a vast and growing body of machine learning studies has established that machine systems, especially those that emulate the essential computational architecture and functionalities of neural systems, can deep-learn to perform a variety of tasks from suitably labeled examples,^39^^,^^105^^–^^107^ including medical images.^108^^,^^109^ A considerable body of cognitive scientific studies has shown that biological organisms, including humans, can learn task-relevant information from examples. Previous work has shown that pigeons can be trained to reliably detect cancer in medical images.^44^ It is important to note that, while few previous studies of biological learning have explicitly referred to the learning methodology as DL, and there is some academic debate about whether biological learning can ever truly qualify as DL (see, e.g., Refs. 43, 110, and 111), it is clear that such learning is, for all practical purposes, indeed DL.^37^ Thus, the principle that complex visual patterns, including those in medical images, can be deep-learned from examples has been well-established from previous studies, and is not novel to our study. Rather, what is new about our study is that it leverages many lines of previous work to establish the outlines of a fairly effective methodology for training human subjects to detect certain cancers in mammograms. That is, it shows that DL methods can be used to train naïve, nonprofessional subjects to recognize diagnostic visual patterns of certain cancers, specifically microcalcifications and breast masses, in mammograms. In doing so, it also helps highlight the fact that expertise in visual pattern recognition can be acquired without having to first acquire medical expertise, i.e., development of visual pattern recognition expertise is dissociable from the development of medical expertise *per se*, which has important implications for medical education (see below). Thus, the present study identifies DL as a principled and potentially highly effective method for addressing the aforementioned lack of well-established methods of developing perceptual expertise in fields like radiology and pathology. Our study also identifies a few additional potential strategies, such as opportunities to review perceptual decisions in light of feedback, that may help improve DL performance and demonstrates the potential methodological efficacy of RSA for quantitatively assessing aspects of this expertise development.

For the reasons outlined above, our studies focused exclusively on mammograms with microcalcifications and breast masses. The aforementioned fact that these two cancers did not differ significantly in the DL effects they produced (data not shown) provides some limited evidence, but by no means proves, that the DL effects we report can generalize across different types of breast cancers. It, therefore, stands to reason—again, in principle—that this methodology may generalize to other types of breast cancers or other types of medical images, provided that the underlying visual patterns were fairly visually clear-cut and reasonably consistent across a given set of training images, i.e., provided there are statistical visual pattern/s that, individually or together, can distinguish cancerous mammograms from healthy ones (also see below). After all, this is the implication of the principles of statistical learning in machines and humans,^34^^,^^112^^–^^116^ and the present study demonstrates this applies at least in principle to mammograms. For the same set of reasons, our results also straightforwardly suggest that our methodology is potentially useful, upon further development and testing, in the future for aspects of medical education and testing that involve recognition of diagnostic visual patterns^117^^–^^120^ (also see below).

### 4.2 Some Important Caveats

In addition to the various caveats noted in context above, a few important ones are worth highlighting here. First, the present study shows only that principles of deep learning can be used to train naïve subjects in certain types of medical image perception tasks, and not that they can be used in all aspects of training that involve medical images, nor that the training procedures we describe are sufficient to fully train subjects to recognize all diagnostic patterns in all types of medical images, nor that this is necessarily how practicing radiologists learn to detect visual patterns of cancer (also see below). Moreover, some of our sample sizes (especially those in experiments 3 and 4) were, out of practical constraints, smaller than optimal. For these reasons, we re-emphasize that our study should be considered a proof-of-principle study rather than a standardization of the underlying DL training protocol. Indeed, we believe that every facet of our methodology can be substantially improved upon, including but not limited to stimulus selection, stimulus presentation, feedback and response review methods, etc.

It is also important to emphasize that it remains unclear whether *all* naïve subjects can be successfully trained using our methodology, since the four subjects who voluntarily discontinued their participation in the study (see Sec. 2) were performing below the criterion at the time (data not shown). While it remains possible that they may have learned the task to criterion in due course, it also remains possible that they may not have.

It is also worth pointing out that our study uses a relatively easy case to make our point. We used mammograms with microcalcifications and breast masses as training examples, which are “easy” in three main respects: First, microcalcifications and breast masses tend to have a visually salient “speckled” or “clumped” appearance,^54^^,^^55^^,^^121^^–^^125^ respectively, that is relatively easily to recognize.^1^^,^^3^^,^^126^ That is, as breast cancers go, the diagnostic visual patterns in microcalcifications and breast masses are among the easiest to recognize. Similarly, malignant *vs*. benign breast masses also tend to have visually salient distinguishing features (see, e.g., Refs. 127–130). However, the patterns in other breast cancers tend to be considerably subtler, more abstract and/or variable.^131^ Second, microcalcifications and malignant breast masses can, to a first approximation, be recognized without any background knowledge, such as the underlying breast anatomy, an understanding (however implicitly) of the generative model of mammograms, etiology of breast cancer, etc. A third, related point is that in many other types of breast cancer, such as inflammatory breast cancer,^132^^–^^136^ visual information plays a far less decisive role in cancer diagnosis than in case malignant microcalcifications or breast masses. In sum, there is more to clinical diagnosis of breast cancer than recognizing complex visual patterns, and there is more to being an expert radiologist than just the perceptual expertise in recognizing subtle image patterns. Our study, by design, addressed one specific aspect of the complex task of breast cancer diagnosis, namely the perception of diagnostic visual patterns.

### 4.3 Future Directions

To the extent that our methods result, at best, in $d^{\prime }$ values in the range of 2.5 to 3, there is self-evidently much room for improving to achieve higher $d^{\prime }$ values. It is possible that combining purely sensory learning with medical training will improve performances over and above what we were able to achieve in the present study. After all, this is roughly how radiologists and pathologists learn their craft, although our methods provide a principled method for training subjects in the diagnostic visual patterns.
